# Structured light enhanced entoptic stimuli for vision science applications

**DOI:** 10.3389/fnins.2023.1232532

**Published:** 2023-07-25

**Authors:** Dmitry A. Pushin, David G. Cory, Connor Kapahi, Mukhit Kulmaganbetov, Melanie Mungalsingh, Andrew E. Silva, Taranjit Singh, Benjamin Thompson, Dusan Sarenac

**Affiliations:** ^1^Department of Physics, University of Waterloo, Waterloo, ON, Canada; ^2^Centre for Eye and Vision Research, Hong Kong, Hong Kong SAR, China; ^3^Institute for Quantum Computing, University of Waterloo, Waterloo, ON, Canada; ^4^Department of Chemistry, University of Waterloo, Waterloo, ON, Canada; ^5^School of Optometry and Vision Science, University of Waterloo, Waterloo, ON, Canada; ^6^Department of Physics, University at Buffalo, State University of New York, Buffalo, NY, United States

**Keywords:** Haidinger's brush, structured light, entoptic phenomena, macular pigment, age-related macular degeneration

## Abstract

The dichroic macular pigment in the Henle fiber layer in the fovea enables humans to perceive entoptic phenomena when viewing polarized blue light. In the standard case of linearly polarized stimuli, a faint bowtie-like pattern known as the Haidinger's brush appears in the central point of fixation. As the shape and clarity of the perceived signal is directly related to the health of the macula, Haidinger's brush has been used as a diagnostic marker in studies of early stage macular degeneration and central field visual dysfunction. However, due to the weak nature of the perceived signal the perception of the Haidinger's brush has not been integrated with modern clinical methods. Recent attempts have been made to increase the strength of the perceived signal by employing structured light with spatially varying polarization profiles. Here we review the advancements with the structured light stimuli and describe the current challenges and future prospects.

## 1. Introduction

### 1.1. Age-related macular degeneration and the Haidinger's brush

Age-related macular degeneration (AMD) is a global leading cause of irreversible blindness (Lim et al., 2012). Deposition of numerous subretinal drusen is known to be an early sign of AMD preceding the intermediate stage of the disease, which typically involves central field distortions and impairment of visual acuity (Bowes Rickman et al., 2013; Wong et al., 2022). Further degeneration of the retina and choroidal neovascularization (CNV), and the proliferation of small extraneous and fragile blood vessels within the choroid, occur during advanced AMD (Yeo et al., 2019; Borrelli et al., 2020). If the early functional signs of macular degeneration can be detected, clinically visible anatomical damage to the eye can be more readily prevented or minimized (Heesterbeek et al., 2020; Di Carlo and Augustin, 2021). Consequently, detecting AMD at the earliest stage is invaluable. Current methods to detect AMD include visual identification of the drusen and CNV using a slit lamp and imaging the retina with optical coherence tomography (OCT) (Cook et al., 2008; Waldstein et al., 2020). Unfortunately, the clinical manifestations of an early stage of AMD are subtle, and the disease is often detected after noticeable visual impairment has begun (Green et al., 1985; Bowes Rickman et al., 2013).

A promising diagnostic marker for detecting the early signs of AMD may be the perception of entoptic phenomena when viewing polarized blue light (Forster, 1954). Uniformly polarized blue light stimuli induce a bowtie-like entoptic pattern known as the Haidinger's brush. The discovery of the Haidinger's brush dates back to 1844 (Haidinger, 1846), and the first mechanism models developed by Maxwell and Helmholz (Maxwell, 1850; von Helmholtz, 2013) postulated the existence of a radial filter in the eye. Later investigations confirmed the presence of a dichroic macular pigment in Henle fibers in the retina that possess radial arrangement throughout the fovea (Horváth and Varjú, 2004). Although the exact mechanism that is responsible for the Haidinger's brush is still unclear, it is typically attributed to the tangential arrangement of the macular pigment molecules and the radial arrangement of the Henle fibers (Horváth and Varjú, 2004; Le Floch et al., 2010; Misson et al., 2015, 2019; Misson and Anderson, 2017; Wang et al., 2022). The relevant dichroic macular carotenoids, namely lutein, zeaxanthin, and meso-zeaxanthin possess an anisotropic absorption peak at approximately 460 nm (Temple et al., 2015; Mottes et al., 2022). Their placement in the radially oriented fibers effectively forms a weak radial polarizer in the human eye for the color blue.

Haidinger's brush has been employed as a diagnostic marker in studies of age-related macular degeneration (Forster, 1954; Naylor and Stanworth, 1955; Müller et al., 2016; Misson et al., 2020, 2021). A major focus is on determining the time period between polarization-based vision loss and normal vision loss in people suffering from AMD. However, despite the developments associated with the Haidinger's brush, modern clinical tools do not employ entoptic phenomena for diagnosing AMD. One of the major reasons being the faint nature of the entoptic signal. The recent integration of a structured light toolbox into vision science aims to address this problem by greatly enhancing the visibility and versatility of entoptic phenomena.

### 1.2. Development of structured light techniques

The development of custom light fields or “structured light” has seen remarkable progress in the last 30 years (Chen et al., 2021; Ni et al., 2021; Bliokh et al., 2023). The core idea is to induce non-trivial propagation properties by tailoring the light beam's wave front. For example, imprinting an azimuthally varying phase profile creates orbital angular momentum (OAM) states that possesses a helical wavefront and carry quantized OAM (Bazhenov et al., 1990; Allen et al., 1992); imprinting a cubic phase profile creates the Airy beams that possess a curved trajectory in free space and self-healing property whereby the beam appears to reconstruct itself in the presence of obstacles (Berry and Balazs, 1979); and imprinting a radial phase prepares the “non-diffractive” Bessel beams (Indebetouw, 1989). The enabling properties of structured light beams and the access to new degrees of freedom have brought forth a wide range of impactful applications in optical phenomenology and microscopy, high-bandwidth communication, manipulation of matter, and quantum science (Mair et al., 2001; Andersen et al., 2006; Marrucci et al., 2006, 2011; Maurer et al., 2007; Padgett and Bowman, 2011; Wang et al., 2012; Ritsch-Marte, 2017; Sarenac et al., 2018; Schwarz et al., 2020; Cameron et al., 2021).

Numerous methods for preparation and characterization of structured light beams have been developed (Rubinsztein-Dunlop et al., 2016). However, the single major technological driving force responsible for the wide adaptation of the structured light techniques is the development of an optical component called the Spatial Light Modulator (SLM) (Curtis et al., 2002). The SLM is capable of imprinting an arbitrary 2D phase profile over the input beam, thus enabling the preparation of complex phase and intensity structured beams. The ability of modern SLM devices to accomplish this with fast switching rates and high resolution further increases their applicability.

The coupling of polarization to structured light enables the preparation of beams with spatially varying polarization profiles (Marrucci et al., 2011), opening avenues to applications in high-bandwidth communication and optical metrology (Milione et al., 2015; Rubinsztein-Dunlop et al., 2016). These states were also the backbone of the recent integration of structured light techniques into vision science for the creation of stimuli with higher numbers of azimuthal fringes (Sarenac et al., 2020), enabling the perception and discrimination of Pancharatnam-Berry phases (Sarenac et al., 2022), measuring the visual angle of entoptic phenomena (Kapahi et al., 2023), retinal imaging using structured light (Kapahi et al., 2023), and the creation of radially varying entoptic stimuli (Pushin et al., 2023).

## 2. Structured light for vision science applications

The typical action of a SLM is to induce an arbitrary spatially dependant phase profile *f*(*x, y*) onto the polarized input beam (typically horizontal):


(1) $$\begin{array}{l} |\Psi_{SLM}\rangle =e^{if(x,y)}|H\rangle , \end{array}$$

where the resolution of *f*(*x, y*) is set by the pixel size of the SLM, typically a few microns in size. Each pixel can be individually addressed to set the phase at its location between 0 and 2π, and the fast switching rates of the SLM enable one to vary *f*(*x, y*) in real time. In the case of vision science the focus has been on creating spatially varying profiles of linear polarization. The action of the human eye can be modeled as a radial polarization filter, and therefore, it is convenient to consider phase profiles with radial and azimuthal symmetry. With an appropriate input and subsequent beam manipulation the general state of the structured light beams in recent vision science studies can be expressed as:


(2) $$\begin{array}{l} |\Psi_{SL}\rangle =\frac{1}{\sqrt{2}}\left[e^{i(n_{r}r+\ell \phi +\theta t)}|R\rangle +|L\rangle \right], \end{array}$$

where (*r*, ϕ) are the transverse spatial coordinates, *n*_*r*_ and ℓ and the radial and OAM numbers, |*L*〉 and |*R*〉 are the right and left circularly polarized states, and θ*t* is a time varying phase shift that dictates the speed of the perceived entoptic motion. Sarenac et al. (2020) showed that the number of entoptic azimuthal fringes that a human sees when viewing optical states with a superposition of right and left circular polarization coupled to two different orbital angular momentum (OAM) values (ℓ_1_ and ℓ_2_) is equal to the number (*N*) of radial lines in the corresponding polarization profile of the beam, where *N* = |(ℓ_1_ − ℓ_2_) − 2|.

A new challenge that arises with the structured light stimuli is taking into account the effect of free space propagation which alters the beam profile. In the case of OAM beams, a black obstruction region naturally arises in the middle as the beam propagates. This feature was present in Sarenac et al. (2020). To remove the effects of free-space propagation, a technique can be implemented to image the plane of the state preparation onto the retina (Kapahi et al., 2023). This is analogous to a microscopy 4f imaging system, whereby the state at the location of the SLM is imaged at the location of the retina. The decoupling of free space propagation has the additional benefit of enabling the use of precise arbitrary obstructions. For example, the middle region can be intentionally obstructed to test the threshold of polarization-based peripheral vision (Kapahi et al., 2023). An interesting result of Kapahi et al. (2023) study is that the perceived size of the entoptic pattern with *N* = 11 azimuthal fringes was 9.5°±0.9°. This significantly differs from previous estimates of the Haidinger's brush phenomenon's extant (*N* = 2 azimuthal fringes), of 3.75° (Coren, 1971), suggesting that higher azimuthal fringe density increases pattern visibility.

**Figure 1:**
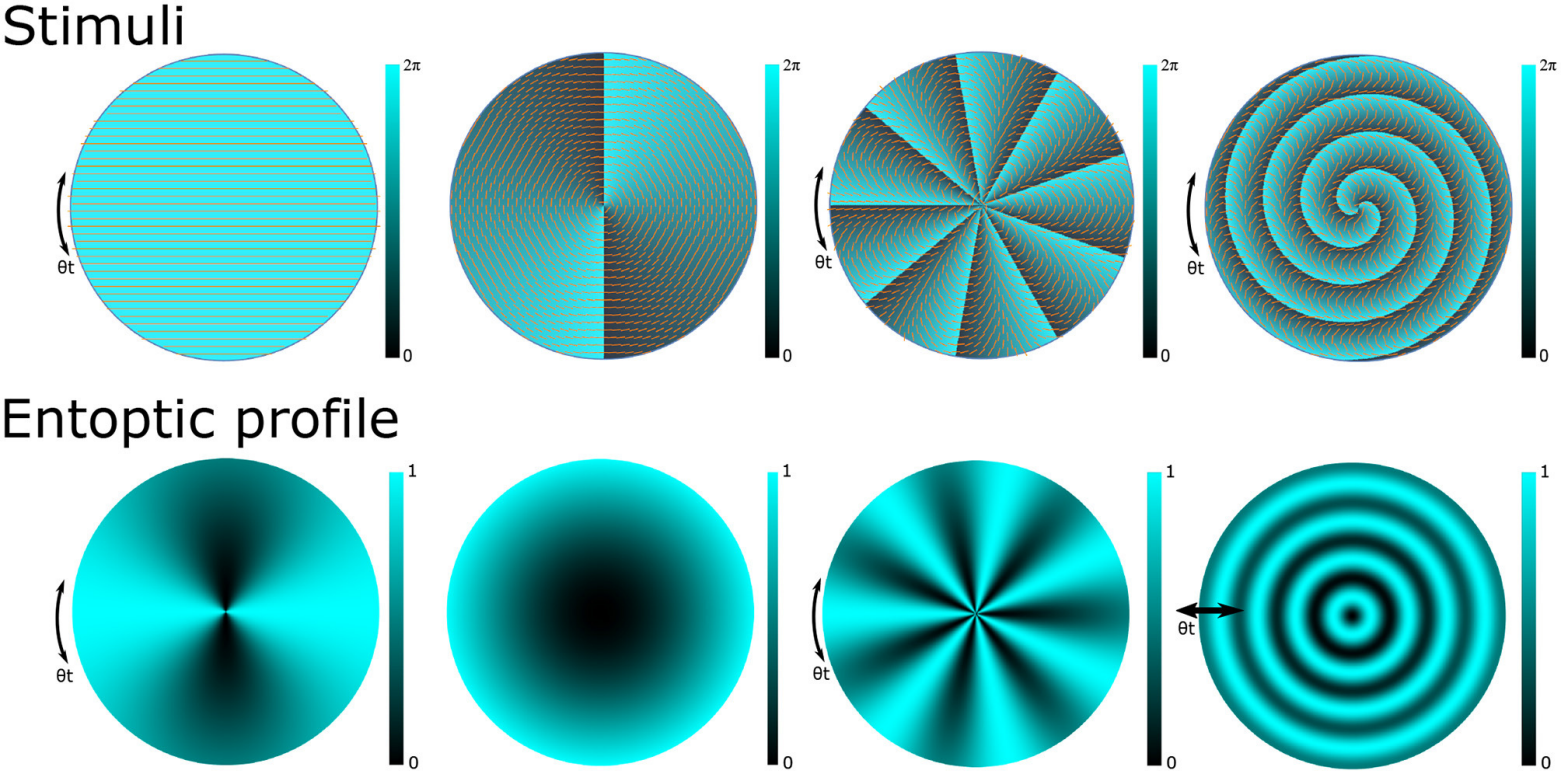
Examples of phase and polarization profiles of structured light stimuli **(top)** and the corresponding entoptic profiles that a participant with a healthy macula would observe **(bottom)**. The clarity of the entoptic profiles is proportional to macular pigment density which typically peaks at the central point of vision and decreases with eccentricity. The first column depicts a horizontally polarized light stimulus and the Haidinger's brush. Introducing structured light techniques to prepare stimulus with polarization coupled orbital angular momentum (OAM) states allows us to induce a wide variety of entoptic patterns. The second column depicts the scenario where the stimulus with OAM = 2 is used to match the structure of the Henle fibers thereby inducing a monotone entoptic pattern. The third column depicts the use of higher OAM numbers to induce stronger stimulus with higher numbers of azimuthal fringes. The last column depicts the use of a radial state coupled to an OAM = 2 state that induces entoptic profiles with radially varying fringes.

A multitude of novel perception tasks are enabled with the structured light stimuli. Several examples of stimuli and their corresponding entoptic phenomena are shown in Figure 1. The first column shows the case of (*n*_*r*_ = 0, ℓ = 0) that results in a uniformly polarized stimulus and the perception of the Haidinger's brush pattern described earlier. The second column considers a stimulus whose polarization profile matches the orientation of the eye's radial filter, resulting in a uniform entoptic profile. The third column considers a stimulus with polarization coupled to a higher OAM state (*n*_*r*_ = 0, ℓ = 9) resulting in an entoptic profile of *N* = 7 azimuthal fringes. The last column considers a stimulus with a OAM = 2 coupled radial state (*n*_*r*_ > 0, ℓ = 2) whereby the OAM = 2 decouples from the radial filter of the eye and the resulting entoptic profile is along the radial direction as shown. Pushin et al. (2023) employed this stimulus to test discrimination sensitivity to inwards and outwards radial motion. It was found that participants had more difficulty discriminating radial motion directions than rotational motion directions. A possible cause could be that in comparison to azimuthally varying stimuli where the fringe oscillations are along the direction of constant macular pigment, radially varying entoptic motion is along the direction with the most change in macular pigment (Pushin et al., 2023).

A major challenge in perception tasks involving uniformly polarized light stimuli and the Haidinger brush is compensating for the ocular birefringence that is oriented about a roughly horizontal axis and subjectively varies in magnitude (Van Blokland and Verhelst, 1987; Bour, 1991; Knighton and Huang, 2002). For some values of birefringence the rotation of the Haidinger's brush becomes undetectable while for others it can appear to rotate in the opposite direction. Kapahi et al. (2023) showed that for structured light states with ℓ > 3, the perceived rotation direction of the entoptic phenomenon is insensitive to ocular birefringence.

**Figure 2:**
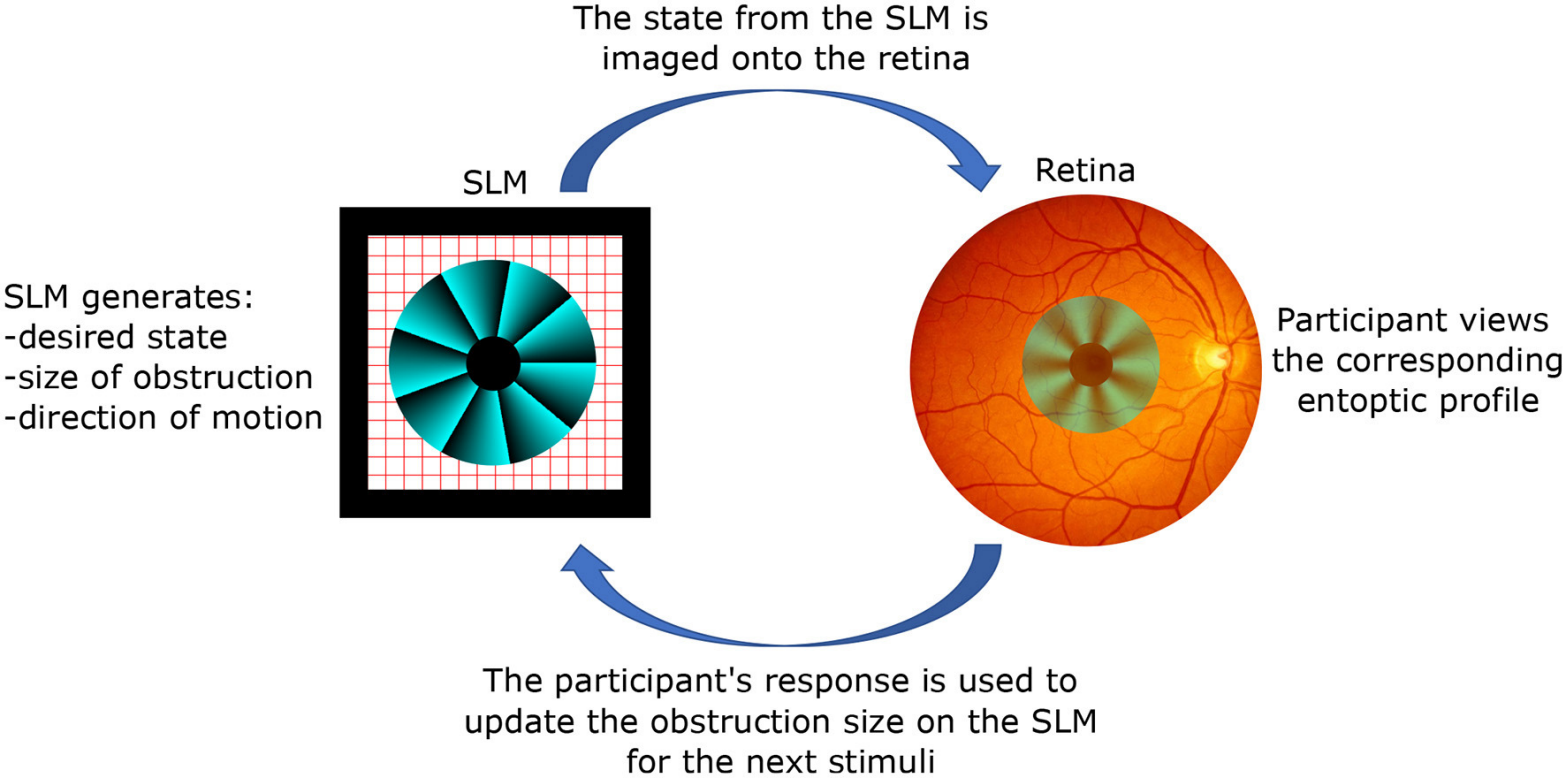
The working principle of the studies with structured light stimuli. A spatial light modulator (SLM) creates an arbitrary polarization state with spatial resolution limited by its pixel size (modern values around ≈ 3 μ*m* by 3 μ*m*). Given the versatility of the SLM, one can introduce arbitrary obstructions, such as the depicted example which removes the central region in order to test the participant's peripheral vision. Optics components (not shown) are used to project the state from the location of the SLM to the participant's retina, thereby removing propagation effects. The size of the obstruction can be varied according to the participant's feedback, and a threshold value for eccentricity can be obtained through a standard staircase method.

A typical setup and procedure for perception tasks with structure light stimuli is depicted in Figure 2. The SLM prepares the desired state for observation, which is then imaged onto the participant's retina. Depending on what is being tested, an obstruction may be introduced onto the phase profile. The participant views the corresponding entoptic profile and performs a discrimination task, for example, discriminating the direction of motion. Depending on the response, the SLM updates the obstruction size for the next stimulus. A reliable psychophysical threshold can then be obtained using a staircase method where the size of the obstruction is varied according to the accuracy of the participant's responses (Kapahi et al., 2023; Pushin et al., 2023).

## 3. Future prospects

Several exciting avenues can be directly explored given the advances in preparation of structured light stimuli. For example, whereas Kapahi et al. (2023) determined that the perceived size of the entoptic pattern with *N* = 11 azimuthal fringes was 9.5° compared to the Haidinger's brush phenomenon's extant of 3.75°, a study to quantify the relationships between the number of azimuthal fringes and perceived size has not yet been done. Similar opportunity is available for the studies with radial numbers (Pushin et al., 2023). Having the ability to determine the apparent size vs. the density of fringes will enable a tomographic reconstruction of the macular pigment profile that is responsible for the polarization-based perception. Furthermore, given that the extent of the structured light induced entoptic images is shown to extend beyond the regions of the fovea, a study is needed to determine the relationship between the perceived size and the thickness of the retinal fiber layers.

The studies with structured light stimuli up to now have been performed with participants that possess a healthy macula. The application of these methods to participants that are at various stages of AMD has not yet been reported. Although the studies with an obstruction present have been done with a central obstruction (Kapahi et al., 2023; Pushin et al., 2023), when considering participants with AMD a more appropriate obstruction will have to be devised as those participants might already have a problem with their central field of vision.

Although Kapahi et al. (2023) introduced retinal imaging using structured light, this was only in terms of intensity images that were used to determine the exact visual extent of the perceived entoptic phenomenon. It may be possible to extend structured light retinal imaging to directly quantify the polarization content of the reflected light in order to associate retinal structural features with polarization sensitivity and to assess macular health without any need for participant interaction.

In addition to the AMD related applications, structured light stimuli also enable several interesting physics applications in the rising field of quantum vision (Loulakis et al., 2017; Margaritakis et al., 2020; Gassab et al., 2023). Sarenac et al. (2022) tested the ability of human observers to discriminate distinct profiles of spatially dependant geometric phases when directly viewing stationary structured light beams. Participants used self-generated eye movements to induce motion in the perceived entoptic phenomenon. Given the access to the additional OAM degree of freedom, an interesting future experiment to consider for structured light stimuli is the measurement of multi-partite correlations with human detectors performing polarization-based Bell-state projections (Shen et al., 2021).

## 4. Conclusion

Technological advancements in preparation and characterization of structured light have been successfully integrated into vision science applications. This young and exciting field contains many opportunities to gain additional insight into macular health by integrating structured light, quantum optics, and vision science. For example, larger and more visible entoptic percepts can be created than with traditional Haidinger's brush, and obstructions with varying sizes can be introduces to determine interpretable thresholds.

## Author contributions

All authors contributed to the writing and editing of the manuscript.
